# Effects of on-site Supportive Communication Training (On-site SCT) on doctor-patient communication in oncology: Study protocol of a randomized, controlled mixed-methods trial

**DOI:** 10.1186/s12909-024-05496-x

**Published:** 2024-05-10

**Authors:** KK Antonsen, AT Johnsen, LØ Poulsen, JD Lyhne, L Lund, S Eßer-Naumann, S Timm, LH Jensen

**Affiliations:** 1 Department of Oncology, Vejle Hospital, University Hospital of Southern Denmark, Vejle, Denmark; 2 Department of Regional Health Research, University of Southern Denmark, Vejle, Denmark; 3 Department of Oncology and Palliative Care, Zealand University Hospital, Roskilde/Naestved, Denmark; 4 Department of Oncology, Aalborg University Hospital, Aalborg, Denmark; 5 Center for Shared Decision Making, Vejle Hospital, University Hospital of Southern Denmark, Vejle, Denmark

**Keywords:** Communication skills training, Continuing professional development, Medical education, Job satisfaction, Burnout, Communication, Multidisciplinary teamwork, Oncology

## Abstract

**Background:**

The quality of communication in oncology significantly impacts patients' health outcomes, as poor communication increases the risk of unnecessary treatment, inadequate pain relief, higher anxiety levels, and acute hospitalizations. Additionally, ineffective communication skills training (CST) is associated with stress, low job satisfaction, and burnout among doctors working in oncology. While acknowledging the importance of effective communication, the specific features of successful CST remain uncertain. Role-play and recorded consultations with direct feedback appear promising for CST but may be time-consuming and face challenges in transferring acquired skills to clinical contexts. Our aim is to bridge this gap by proposing a novel approach: On-site Supportive Communication Training (On-site SCT). The concept integrates knowledge from previous studies but represents the first randomized controlled trial employing actual doctor-patient interactions during CST.

**Methods:**

This randomized multicenter trial is conducted at three departments of oncology in Denmark. Doctors are randomized 1:1 to the intervention and control groups. The intervention group involves participation in three full days of On-site SCT facilitated by a trained psychologist. On-site SCT focuses on imparting communication techniques, establishing a reflective learning environment, and offering emotional support with a compassionate mindset. The primary endpoint is the change in percentage of items rated “excellent” by the patients in the validated 15-item questionnaire Communication Assessment Tool*.* The secondary endpoints are changes in doctors’ ratings of self-efficacy in health communication, burnout, and job satisfaction measured by validated questionnaires. Qualitative interviews will be conducted with the doctors after the intervention to evaluate its relevance, feasibility, and working mechanisms. Doctors have been actively recruited during summer/autumn 2023. Baseline questionnaires from patients have been collected. Recruitment of new patients for evaluation questionnaires is scheduled for Q1-Q2 2024.

**Discussion:**

This trial aims to quantify On-site SCT efficacy. If it significantly impacts patients/doctors, it can be a scalable CST concept for clinical practice. Additionally, qualitative interviews will reveal doctors' insight into the most comprehensible curriculum parts.

**Trial registration:**

April 2023 – ClinicalTrials.gov (NCT05842083). April 2023 – The Research Ethics Committee at the University of Southern Denmark (23/19397).

## Background

Communication between patients and doctors plays a central role in cancer treatment. Successful and effective communication not only strengthens the doctor‒patient relationship; it also enhances patients’ understanding of the treatment process [1], promotes shared decision making [2, 3], and improves patients’ quality of life [4]. Evidence suggests that individualized and empathic communication plays an important role in patient satisfaction, treatment adherence, empowerment, and in navigating the course of the disease [5].

In the field of oncology, communication is particularly complex due to the serious nature of the disease, the growing number of treatment options, and the often uncertain treatment outcomes with risk of recurrence [6]. To understand each patient’s feelings, ideas, concerns [7], and preferences [8] doctors must tailor their communication, which can be a challenging task [9].

Dissatisfaction with perceived communication is a frequent cause of patient complaints [10], and it likely occurs more frequently than officially reported [11]. Poor communication correlates with negative patient experiences and is associated with outcomes such as insufficient pain relief, reduced commitment to treatment decisions, higher risk of unnecessary treatments, increased anxiety levels, and more frequent acute hospitalizations [12, 13]. Analyzing the implications of inadequate communication in cancer care is highly complex, and the potential costs can be substantial, encompassing economic, social, and psychological burdens for both individuals and society [12].

Among doctors working in oncology, inadequate training in communication is correlated with distress, reduced job satisfaction, and emotional burnout [1, 14]. Clearly, this can lead to significant implications for the individual doctor, but overall, exhausted healthcare staff will provide poorer treatment and care [15, 16].

### Features of effective communication skills training

Evidence suggests that communication skills can be taught [17], but uncertainty remains as to the specific components contributing to successful communication skills training (CST) [6]. This is due to the diverse CST intensity, formats, and contents of existing studies along with the wide range of outcome measures used, which makes it difficult to conclude on the results.

When Bos-van den Hoek et al. [18] in 2019 synthesized findings from reviews of the past decade, including a Cochrane review from 2018 [6], certain elements emerged as potentially important factors in the design of CST interventions. Programs spanning over three days (24 h) appeared more effective than shorter programs [17], and post-training follow-up showed potential significance [19]. Training involving active skills practice with feedback on observed situations with real or simulated patients seemed more efficient [20, 21].

Role play with peers or actors combined with recorded consultations has been used in CST, but no trials used real patients during on‐site training [6]. Studies on CST with recorded consultations/role-play have shown a positive effect on key communication skills [22] and increased self-efficacy [23]. Two studies have demonstrated long-term maintenance of acquired skills [24, 25]. Until now, no effect on burnout has been demonstrated [26, 27].

### Psychologists may play an important role in a learner-centered CST approach

As described, communication skills training can hold many different formats. In the Cochrane review, most trials specified the use of “learner‐centered, experiential, adult education methods” conducted by experienced facilitators [6]. Training facilitated by a psychologist allows for involvement of critical elements in relationships and establishment of alliances with the patients [28]. In addition, a psychosocial foundation can facilitate a learner-centered setting prioritizing the needs and agendas of the trainees [29]. This creates a safe environment for educational and contemplative progression to take place.

### Research gap and study objectives

Until now, studies in CST have relied on role-playing, recorded conversations, group discussions or didactic teaching [6]. These approaches are time-consuming for the doctor, and the transfer of communication skills learned in a training environment may be challenging [30, 31].

To bridge this gap, we have developed a supportive communication training invention taking place in the daily clinic (On-site SCT) based on the current knowledge of effective CST. In the present study, trained psychologists facilitate the training in the outpatient clinic, thereby merging medical expertise with psychological insights. This innovative approach offers several potential advantages in terms of feasibility and efficiency. On-site SCT has the potential to facilitate direct skill transfer, provide personalized training, and promote interdisciplinary collaboration to address both physical and mental health aspects—all while the doctor remains in service.

## Hypotheses

- Patients’ ratings of doctors’ performance in the area of interpersonal and communication skills will increase when doctors participate in On-site SCT.
- Participation in On-site SCT will increase the doctors’ self-evaluation in relation to communication efficacy and job satisfaction, and decrease their experience of burnout.

## Methods

### Setting

This study is a randomized, controlled, multicenter study with three participating sites: Vejle Hospital, Aalborg University Hospital, and Zealand University Hospital. Hypotheses are tested by comparing questionnaires before and after the intervention.

Each site has a study coordinator to ensure adherence to the intervention protocol. The project employs seven experienced psychologists (one person-year in total).

## Participants

### Patients

Patients attending a doctor's appointment at the outpatient clinic during the data collection periods are eligible to participate in the study. They will be encouraged to complete the Communication Assessment Tool (CAT) [32], a validated questionnaire, providing their evaluation of the doctor’s performance in the areas of interpersonal and communication skills. Each questionnaire is marked with the initials of the specific doctor, allowing for comparison between doctors in the intervention and control groups. When possible, questionnaires are distributed by study nurses or secretaries instead of the doctors themselves to reduce the risk of selection bias (i.e., the situation where doctors select the patients they hand a questionnaire). Questionnaires will be administered at baseline and evaluation. In case some patients do not wish to participate, their empty questionnaires will be collected to track the response rate.

Our aim was to collect questionnaires from all patients interacting with a doctor eligible for enrolment, as this approach will enable us to compare participating and nonparticipating doctors. Baseline questionnaires have been collected at all three sites during summer/fall 2023. The recruitment of new patients for evaluation questionnaires is scheduled for Q1-Q2 2024.

### Doctors

**Fig. 1:**
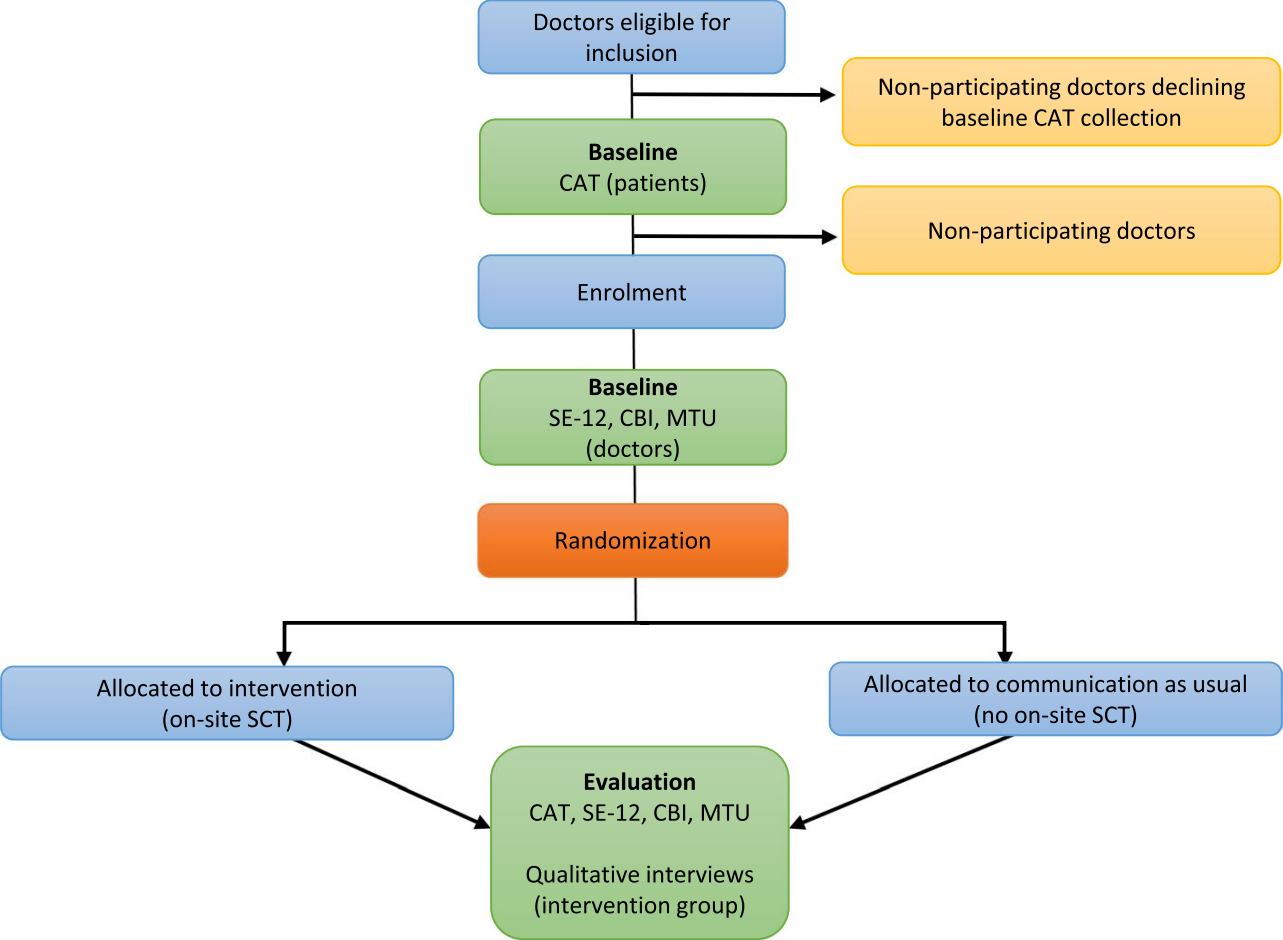
Study flowchart*.* CAT: Communication Assessment Tool, SE-12: Self-efficacy in Health Communication; CBI: Copenhagen Burnout Inventory; MTU: Medarbejdertilfredshedsundersøgelse (employee satisfaction survey)

The doctors in this clinical trial are either in specialty training or are board-certified specialists in clinical oncology. All doctors employed at the three participating sites working at least 4 days/month in the outpatient clinic undergo screening for eligibility. The cutoff ensures study relevance by including doctors with diverse roles, from doctors with frequent patient contact to doctors with fewer interactions, mirroring real-life hospital scenarios. All participating doctors must provide an informed consent. Doctors whose contract expire during the study period or who are otherwise not able to participate in the full study period, e.g. due to maternity leave, are not eligible. Doctors who are not willing to participate are classified as nonparticipating. All doctors in this group are asked if they are willing to accept baseline collection of CAT questionnaires. A study flowchart is shown *in *Fig. 1.

Doctors have been actively recruited during summer (Vejle Hospital) and autumn (Aalborg and Zealand University Hospitals) 2023.

### Withdrawal criteria

Doctors are free to withdraw from the trial at any time without providing a reason. All dropouts will be registered.

### Randomization

Doctors are randomly assigned to the intervention and control groups in a 1:1 ratio using Research Electronic Data Capture (REDCap) version 13.1.25 and a block size of 2/4. Stratification based on location is implemented to ensure an equal distribution of workload among the psychologists. Stratification will also allow for site comparison in the analysis. The intervention will last 3–4 months at each site, depending on the number of participating doctors.

## Sample size calculation

Approximately 80 out of the around 90 eligible doctors (89%) are expected to participate in the study.

To achieve a power of 80%, a sample size of 2,080 patients (1,040 in each group) will be obtained by sampling 40 clusters (doctors) with 26 patient questionnaires in each group. In total we thus aim to collect 4160 questionnaires. The proportion in the intervention group is assumed to be 0.28 under the null hypothesis and 0.34 [33] under the alternative hypothesis assuming a difference between the group proportions of 0.06. Due to the lack of international consensus in this research area, an absolute change of 6% was agreed upon by mutual consensus in the steering group, as we believe such change would represent a clinical relevant difference for the patients. The proportion in the control group is assumed to be 0.28. The sample size calculation is based on a two-sided Z Test (unpooled) with intracluster correlation (ICC) set at 0.005, and significance level of the test at 0.05.

Each cluster size of 26 completed questionnaires per doctor at baseline and evaluations aligns with the recommendations from the CAT developers [32]. To meet the target we aim to distribute 30–40 questionnaires per doctor. Staff at each outpatient clinic will assist in the process.

## Intervention

A collaborative effort between a group of experienced psychologists and the first author, KKA, who is a medical doctor, led to the development of the intervention manual. Drawing from the literature presented in the introduction, the intervention was constructed around four elements:

1. *Setting*: The training takes place on-site, which enables context-specific feedback tailored to the clinical scenario and eliminates transfer of acquired skills from a training environment to the workplace [30, 31].
2. *Format/pedagogical tools*: Training is focused on individual objectives defined as learning goals and will be based on direct feedback and reflection [20, 21].
3. *Duration*: The training program lasts three full working days [18]. Although not labeled “follow-up training” [18] the 3–4-week gap between intervention days is designed to foster skill development between sessions.
4. *Facilitation by trained psychologists* [29]: All psychologists are experienced and have read the intervention manual carefully, thereby ensuring fidelity to the intervention. During the intervention period, psychologists receive regular supervision from an experienced psychologist.

### Structure of the intervention days

The psychologists will observe doctor-patient consultations for one full working day at a time (intervention day). At the time of randomization, psychologists are assigned to the same doctors throughout the intervention period. Prior to the first intervention day two one-hour sessions are held by the psychologist for all doctors randomized to the intervention before the first intervention day. The first session is preferably a group meeting where the doctors are introduced to their psychologist, expectations are aligned, and they get acquainted with the term “learning goals”. The second session is preferably individual, where the doctor and the psychologist start the process of formulating one or two learning goal(s).

On intervention days, the psychologist and the doctor agree on a learning goal for the On-site SCT. A time slot of 30 to 45 min is allocated for feedback after completion of three to four consultations, and at the end of the day there is a 60-min session for more thorough feedback and planning. The plan will serve as a guide to refining strategies during the interim period until the next intervention day.

**Fig. 2:**
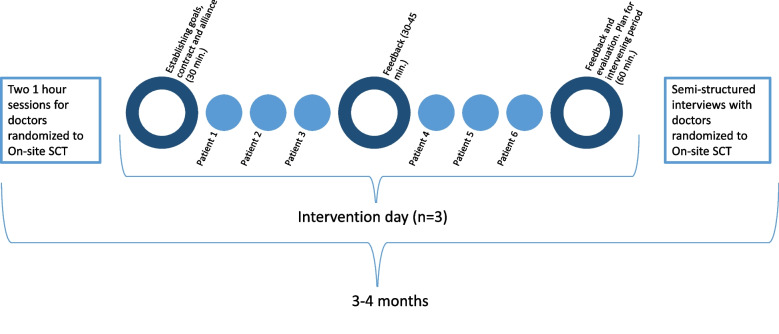
*Overview of the intervention.*On-site SCT: On-site Supportive Communication Training

It is expected that the doctor can be scheduled with six to seven consultations on an intervention day (cf. up to 12 normally). The psychologist will attend the consultations as an observer only. After all intervention days, the psychologist will register data in REDCap, documenting the number of patients seen, time spent on feedback, the psychologist's assessment of the doctor's motivation, and the learning goals set by the doctor. The intervention period for each doctor is 3–4 months as illustrated in Fig. 2.

### Theoretical framework of the intervention

The purpose of the intervention is to contribute to doctors' continuing professional development, focusing specifically on improved doctor-patient communication and increased self-awareness. This will be achieved through reflection on consultation practices aligned with communication knowledge gained from the 3-day On-site SCT.

The intervention manual was designed to cover the following elements: I) *Communication.* Doctors are intended to become better at communicating, including using patient-centered communication focusing on the needs and emotions of the patients. II) *Learning to learn.* Doctors are encouraged to enhance their ability to identify and explore their own learning needs. This process involves formulating “learning goals” and experimenting with new approaches to engage in conversations with patients. III) *Compassion.* Doctors are aimed to gain better insight into their own reactions/emotions and through psychoeducation be introduced to a compassion-based approach to the doctor‒patient relationship and communication.

### Theoretical frameworks of communication

The description of skills for communication with the patients is rooted in the framework of the Calgary-Cambridge Guide [34, 35]. It outlines evidence-based microskills associated with each of the five domains of the consultation process, i.e. initiating the session, gathering information, providing explanations, making plans, and closing the session. The Calgary-Cambridge Guide also describes how to provide structure to the consultation and build a relationship with the patient.

### Theoretical frameworks of learning

**Fig. 3:**
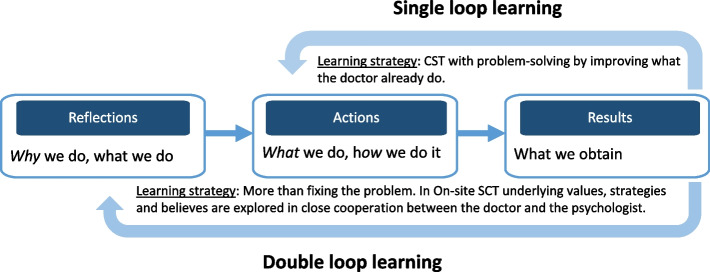
Single vs. double looped learning

This part is grounded in theories of action research [36], which itself builds on action learning, a pedagogical concept asserting that individuals learn most effectively when taking part in formulating what they would like to learn and when tackling real-time problems within their own work environment [37]. Action research involves an additional layer of awareness that facilitates the deliberate questioning of existing perspectives and interpretations – a process often termed double loop learning as depicted in Fig. 3 [38, 39]. It explores how individuals can benefit from reflecting on assumptions, actions, and underlying values, often eliciting tacit knowledge [40].

**Fig. 4:**
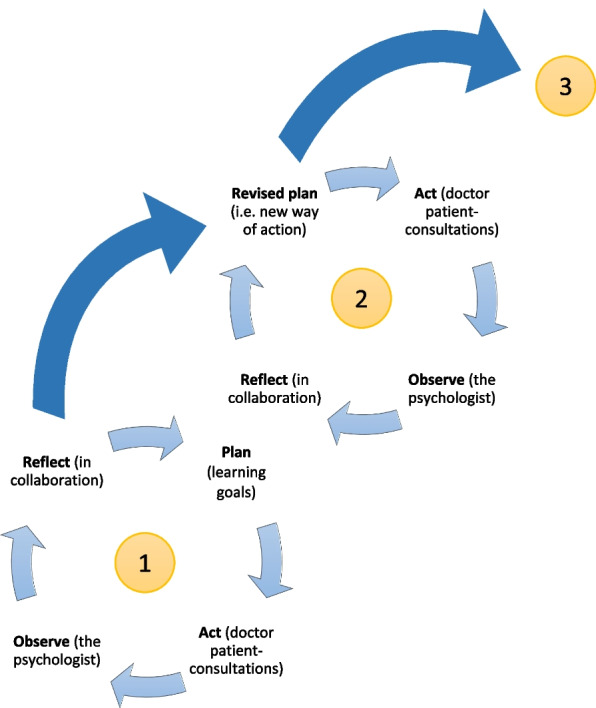
Learning cycles in On-site Supportive Communication Training

As illustrated in Fig. 4 (adapted from Altrichter et al. [36]), the initial sessions (group and individual) in On-site SCT enable doctors to define their initial *learning goals* (Cycle 1). Through the psychologist's observation of the doctor's interaction with the patient, a collaborative reflection opportunity emerges. The joint reflection aims to deepen the doctors' comprehension of their practices (double-loop learning) and, if applicable, to develop novel approaches and strategies to be tested in a subsequent learning cycle.

The project design facilitates different levels of reflection [41]:

- Reflection-on-action occurs after an event to assess actions and outcomes.
- Reflection-for-action focuses on improving future practices.
- Reflection-in-action takes place during an event, enabling real-time adjustments.

During intervention days, most reflections are likely reflection-on-action and reflection-for-action, where psychologists and doctors reflect on specific doctor-patient interactions to establish a revised plan.

The period of 3–4 weeks between intervention days allows for practitioners to engage in reflection-in-action. As described by Schön [42], this reflective practice involves the dynamic assessment and adjustment of one's actions in response to evolving insights during a given situation. It essentially entails a form of metacognition, where individuals actively monitor and adapt their actions based on their developing understanding of the context. According to Schön, the process is most impactful when it arises in response to disruptions in established patterns, which can occur when doctors receive feedback from psychologists regarding communication strategies and routines.

### Theoretical frameworks of compassion

The CST used in the current study is termed "supportive" because all psychologists operate on a compassion-based foundation. Compassion is often defined as “a sensitivity to suffering in oneself and others, with a commitment to try to alleviate and prevent it” [43]. Suffering is often present in patients due to their diagnosis, but it can also be present in doctors, for example when they feel inadequate due to lack of time, when the disease is incurable, or when a patient is experiencing a crisis. The doctors do not undergo training in compassion-focused therapy [44] as part of the On-site SCT, but psychologists may provide psychoeducation rooted in the framework of compassion-focused therapy, when relevant for the doctor. The aim is to assist the doctors in their encounters with patients as well as in their role as medical practitioners. In the process of eliciting learning goals and engaging in reflective practices as a component of double-looped learning, compassion-focused therapy plays a significant role in uncovering doctors’ underlying motivations, emotions, and values.

Qualifications of project psychologistsThe psychologists conducting On-site SCT have experience working with patients in an oncology department. Additionally, they are knowledgeable about the Calgary-Cambridge model for effective medical communication and shared decision-making. The psychologists themselves have undergone more than 100 h of supervision during their psychological training. In connection with the project, they participated in a four-day joint training/start-up meeting covering all aspects of the intervention. Furthermore, the psychologists receive collective supervision every three weeks throughout the project period.

### Experiences from a feasibility trial

An initial version of the intervention manual was tested and adapted in a feasibility trial involving four doctors at the Department of Oncology, Herlev Hospital, Denmark. They all participated in one day of On-site SCT conducted by a psychologist (second author). The feasibility and relevance of the intervention was investigated by interviewing the participating doctors. The interviews were conducted by the first author, KKA. The following observations were made during the trial days and interviews:

*Feasibility*: The intervention was feasible within the designated timeframe and available resources. The presence of the psychologist was well received by doctors and patients, and no patients declined the presence of the observer.

*Relevance and adaptation*: All doctors responded positively to the concept of establishing their individual learning goal(s). It became evident that concentrating on one goal at a time was preferable, as a broader focus made the feedback too abstract within the one-day period.

*Overall experience*: The doctors found the intervention valuable and meaningful recognizing its potential in improving their communication skills.

## Primary outcome measure (patients)

The effect of On-site SCT on patients’ evaluation of the doctors’ communication skills is assessed by the 15-item Communication Assessment Tool (CAT). It is a reliable and valid instrument for measuring patient perceptions of physician performance in the area of interpersonal and communication skills [32]. Since its last item, “The doctor’s staff treated me with respect”, is focused on the staff, it will not be included in this study. The questionnaire was validated in 2007 with a sample of 40 doctors from six distinct medical boards [32]. Later it was translated to Danish and validated to assess the perspectives of Danish patients on clinicians' communication skills [33].

## Secondary outcome measures (doctors)

At baseline and evaluation all participating doctors at the three departments are asked to fill out questionnaires measuring communication self-efficacy, degree of burnout, and job satisfaction.

Self-efficacy in Health Communication (SE-12) consists of 12 questions eliciting healthcare professionals’ perceived self-efficacy in communication with patients. The questionnaire was developed and validated by Axboe et al. [45]. Parts 2 and 3 of the “Copenhagen Burnout Inventory (CBI)” assess work and patient-related burnout, respectively. The questionnaire has been validated in seven types of workplaces, including doctors at a somatic hospital [46]. In the Region of Southern Denmark job satisfaction is measured among 25,000 healthcare workers in a survey every other year, thus offering a large amount of comparable data. Five items on job satisfaction are extracted from the survey for the present study.

## Statistical analysis plan

All statistical analyses will be performed in close collaboration with a statistical consultant based on predefined statistical analysis plans. A 95% confidence interval is applied. P values < 0.05 are considered statistically significant. STATA version 18 will be used for all analyses, and all steps of data management, coding and analysis will be logged.

### Primary endpoint

The primary outcome measure is the change in percentage of “excellent” scores measured by the CAT questionnaire on each of the 14 items. All items are answered on a numerical scale from 1 (poor) to 5 (excellent), as well as the option "not relevant". The difference in percentage of "excellent" scores between patients treated by doctors in the intervention and control groups will be analyzed by binary regression using robust variance estimation and taking clustering of patients seeing the same doctor into account. The model will include participating site as a covariate. Due to the randomization and the high number of observations, exchangeability is expected between the patients in the two study groups, and hence, no further covariates are planned in the model. Exchangability will be investigated using standardized differences. A standardized differences > 0.1 indicate imbalance between groups. Effects will be reported on relative and absolute scales as relative risk (RR) and risk differences (RD).

Data will be collected from the patients’ questionnaires on sociodemographic information, years with cancer, disease stage (curable, incurable, unknown), relation to the doctor (first meet or not), and participation or not of relatives at the consultation. Subgroup analysis stratified by site will be performed.

All analyses will be evaluated as intention to treat. The proportion of missing data in the patient questionnaires will be investigated. If the proportion is less than 5%, a complete case analysis will be performed. If missing data exceeds 5%, an imputation analysis will be applied [47].

### Secondary endpoints

*Self-efficacy in health communication*: Change in the mean sumscore of the SE-12 Survey. All items are answered on a numeric scale at 1–10 as well as the option “not relevant”.

*Burnout*: Change in total score for CBI part 2 (seven questions) and part 3 (six questions). All items are answered on a numeric scale from 0 to 4.

*Jobsatisfaction*: Change in the mean sum score of the MTU. All items are answered on a numeric scale 1–7.

Hypotheses will be tested using paired t test in case of normality and Wilcoxon signed rank test in case of non-normality comparing the intervention and control groups.

Data will be collected on the doctors’ age, sex, spoken language at home, years since university degree, job title and prior participation in communication training. Explorative subgroup analysis will be performed stratified by site and doctors’ experience (years since university degree).

A doctor participating in two or more intervention days with a psychologist is considered compliant with the intervention. Doctors’ questionnaires will be evaluated as intention to treat. In case of crossover, per-protocol analysis will be performed as sensitivity analysis. The proportion of missing data in the doctors' questionnaires will be investigated. If the proportion is less than the expected 5%, a complete case analysis will be performed. In case of missing data above 5%, appropriate imputation methods will be applied.

### Analysis of nonparticipating doctors

We aim to analyze baseline CAT data of both participating and nonparticipating doctors. The analysis aims to identify any significant distinctions that might bias the results and hamper generalizability of the study findings. If the number of nonparticipating doctors is fewer than five, we will suspend the analysis due to the risk of personally identifiable data.

## Data management

REDCap will be used for data management. During the study, data will be processed and stored in accordance with applicable legislation (EU GDPR and the Danish Data Protection Act) using REDCap and OPEN Analyze via the OPEN organization, Odense University Hospital, Region of Southern Denmark.

Questionnaires will be completed on paper and entered into REDCap. A data entry manual will be continually evaluated and updated to ensure uniform and consistent data entry across all sites. Data access in REDCap is restricted to project professionals.

## Qualitative analysis of the doctors’ experience with the intervention

After the intervention, qualitative interviews will be conducted with doctors in the intervention group to gain a deeper understanding of the relevance, feasibility, and working mechanisms of the intervention. The interviews will have the Kirkpatrick evaluation model [48, 49] as a framework, ensuring comprehensive coverage of various aspects of the doctors’ experiences and learning outcomes. The insights gathered from these interviews aim to facilitate the scalability of the curriculum to medical staff education and other continuing professional development activities.

The interviews will follow a semi-structured guide covering topics related to the doctors' experiences with On-site SCT. The interview approach will be hermeneutic-phenomenological [50–54] aiming to comprehend the doctors’ lived experiences with On-site SCT. Data will be analyzed using content analysis focusing on themes related to the relevance, feasibility, and working mechanisms of the intervention. An open-minded approach will be maintained to explore topics not initially considered.

## Discussion

### Strengths

A randomized, controlled trial design enhances the internal validity of the study by randomly assigning oncologists to the intervention or control group. This will reduce selection bias and allow for a more robust assessment of the impact of On-site SCT. Additionally, conducting the study at three sites increases the diversity and representativeness of the sample in providing a stronger basis for generalization and demonstrating the intervention's effectiveness across settings.

The on-site approach allows doctors to balance clinical duties (seeing patients) with ongoing, individualized learning. If effective, On-site SCT should be considered a part of a continuing education program. Thus, it should be seen as a viable alternative to an external three-day full-day course in communication skills, where the costs would include not only the doctor's complete absence from clinical duties but also the course fee. Additionally, fostering collaboration between psychologists and oncologists promotes an interdisciplinary approach to patient care.

The primary endpoint of the study, change in percentage of "excellent" scores, is assessed using a validated questionnaire with expected data from more than 4,000 patients. The patient-centered approach adds value to the study by capturing the patients’ perception of effective communication. The study collects both quantitative and qualitative data from the participating doctors to comprehensively evaluate the effect of the intervention.

As described, selection bias may occur, as the doctors who would potentially benefit the most from the intervention might be more likely to decline participation. To address this hypothesis, we aim to collect baseline CAT questionnaires from patients of all eligible doctors enabling comparison of those participating and not participating. As described, the analysis necessitates a minimum of five individuals in the last group. Conducting tthe analysis allows for a better understanding of any differences between the two groups and helps mitigate the potential impact of selection bias.

It is a strength that the intervention has been tested and thoroughly described in a manual (15 pages). It is developed in Danish but can be translated into other languages to enable international dissemination of the concept.

### Limitations

The nature of the On-site SCT intervention does not allows for blinding of the participants or researchers to the group assignment. This may introduce bias and affect the interpretation of the results.

The study relies on voluntary participation of oncologists, which may introduce selection bias. The doctors who choose to participate may have a greater interest or motivation for improving their communication skills, which could affect the generalizability of the findings.

The CAT questionnaire is the only tool used to assess patients' perceptions in this study. It lacks cancer-specific validation in this particular setting, which could be considered a limitation [55]. While the CAT has been tested in various inpatient settings with no evidence of a ceiling effect [33, 56, 57], higher scores and a potential risk of a ceiling effect were observed when tested in family medicine and outpatient settings [32, 58]. If a ceiling effect is observed, we will apply appropriate statistical methods (Tobit models) to account for the data structure.

Long-term evaluation of the intervention is not within the scope of the study.

### Perspectives

Previous studies have suggested that longer lasting, feedback-based training by experienced facilitators might be the most effective way of conducting CST. The present study has the potential to further explore these issues and provide novel insight into the use of on-site training. If On-Site SCT proves to be effective and efficient, it could become a scalable concept for communication skills training in oncology and other healthcare settings.

## Data Availability

Not applicable.

## Abbreviations

CAT: Communication Assessment Tool
CBI: Copenhagen Burnout Inventory
CST: Communication skills training
On-site SCT: On-site Supportive Communication Training
RD: Risk differences
RR: Relative risk
SE-12: Self-efficacy in Health Communication (the number applies to the 12 items in the questionnaire)
